# Microencapsulation of *Lactobacillus reuteri* by Emulsion Technique and Evaluation of Microparticle Properties and Bacterial Viability Under Storage, Processing, and Digestive System Conditions

**DOI:** 10.1002/fsn3.4533

**Published:** 2024-11-11

**Authors:** Forough Teymoori, Sahar Roshanak, Shadi Bolourian, Rassoul Mozafarpour, Fakhri Shahidi

**Affiliations:** ^1^ Department of Food Science and Technology, Faculty of Agriculture Ferdowsi University of Mashhad Mashhad Iran; ^2^ Department of Food Additives, Food Science and Technology Research Institute Research Center for Iranian Academic Center for Education, Culture and Research (ACECR), Khorasan Razavi Branch Mashhad Iran

**Keywords:** emulsion, gum Arabic, *Lactobacillus reuteri*, microencapsulation, whey protein concentrate

## Abstract

In this research, the emulsification method was used to encapsulate *Lactobacillus reuteri* in microparticles of whey protein concentrate (WPC) at different levels (1%, 2%, and 4%) and gum Arabic (GA) at three levels (0/5%, 1%, and 1/5%) and a constant level of sunflower oil (5%). The results showed that emulsions with higher quantities of wall materials exhibited better encapsulation efficiency (67%/57%) and preservation ability at different temperatures, different pH, and presence of 1% bile salt. During the storage time, the droplet size of the emulsion increased more than two times (from 2.2 to 4.6 μm) and the absolute zeta potential of the optimal emulsion decreased (from −19/63 to −16/76 mV). Encapsulating *Lactobacillus reuteri* in the stabilized emulsion with the highest concentration of wall material improved the cells' protection during storage. The study also observed a decline in the number of primary encapsulated live cells in the gastrointestinal tract (from 4/32 to 3/58 Log CFU/mL) after 90 days of storage. In the case of the nonencapsulated sample, the initial live population decreased from 2.8 to 1 Log CFU/mL after 90 days of storage. The electron microscope images showed that the emulsions became unstable after 30, 60, and 90 days of storage, but the microbial cells were still visible in the continuous phase. Overall, encapsulating *Lactobacillus reuteri* using emulsification technique can preserve the probiotics during storage and “in vitro” gastrointestinal digestion.

## Graphical abstract

The emulsification method was used to encapsulate *Lactobacillus reuteri* in microparticles of whey protein concentrate (WPC) and gum Arabic. Higher concentration of wall materials exhibited better encapsulation efficiency (67.57%) and preservation ability. Encapsulation of *Lactobacillus reuteri* using emulsification technique can preserve the probiotics during storage and “in vitro” digestion.

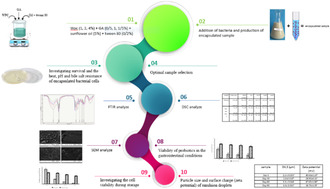

## 1 Introduction

Incorporating beneficial probiotics into food systems can create a sophisticated system that promotes human health and well‐being. *Lactobacillus reuteri* is known to produce a range of antimicrobial compounds, including lactic acid, hydrogen peroxide, reuterin, and reutericycline. These microbial compounds are recognized for their positive impact on the host organism (Azeem et al. 2023). It has been found that *Lactobacillus reuteri* strains cultivated in a laboratory environment are capable of suppressing various intestinal pathogens such as *Escherichia coli*, *Salmonella typhimurium*, *Staphylococcus epidermidis*, *Staphylococcus aureus*, *Helicobacter pylori*, and Rotavirus. In addition, *Lactobacillus reuteri* can produce vitamin B_12_ it has been shown that microencapsulating of *Lactobacillus reuteri* can increases its survival and stability under different conditions. Researches have shown that microencapsulation can improve the stress resistance capacity, storage stability, and colonization ability of *Lactobacillus reuteri*, making it more resistant against harsh conditions in the digestive system and during storage. In recent years, many different microencapsulation techniques have been reported in order to protect probiotics against acids, bile, and other destructive factors that reduce the viability of microorganisms (Islam et al. 2010; Iqbal et al. 2021). Microorganisms are encapsulated in a biodegradable polymer capsule. Many researches have been performed to evaluate the survival rate of microencapsulated microorganisms. A study found that *Lactobacillus reuteri* microencapsulated with a specific mixture of wall materials including soybean protein isolate (SPI) and flaxseed gum, showed increased stress resistance and colonization ability (Wang et al. 2011). Puttarat et al. (2021), also demonstrated that spray‐dried microencapsules fabricated by whey protein isolate and nanocrystalline starch significantly improved the survival of *Lactobacillus reuteri* during long‐term storage, different temperatures, and the digestive system. Consuming microencapsulated *Lactobacillus reuteri* may provide health benefits such as increased probiotic endurance and stability in the gastrointestinal system, as well as better stress resistance and colonization efficiency (Puttarat et al. 2021; Wang et al. 2011). The challenge of maintaining the survival of probiotic bacteria under the harsh conditions of food processing (such as low pH and extreme temperatures) and the acidic and alkaline environments of the digestive system has driven the development of various protective methods (Yao et al. 2020). In recent years, many different microencapsulation techniques have been reported in order to protect probiotics against acid, bile, and other destructive factors that reduce the viability of microorganisms. The common methods of encapsulating probiotics include emulsion formation, extrusion, freeze drying, spray drying, or fluidized bed drying. The above‐mentioned methods have an effect on the characteristics of microcapsules, including dimensions, stability, and encapsulation efficiency (Pech‐Canul et al. 2020; Guo et al. 2024). Researches have shown that microencapsulation techniques, such as spray drying with specific wall material mixtures, can increase the survival rate of *Lactobacillus reuteri*, making it more resistant to acidic and alkaline conditions as well as bile salts. The enhanced survival and stability of *Lactobacillus reuteri* may contribute to the strengthening of the intestinal barrier and potentially other health benefits. Therefore, consuming microencapsulated *Lactobacillus reuteri* may offer advantages in terms of delivering viable probiotics to the intestine and maximizing their potential health benefits (Puttarat et al. 2021; Wang et al. 2011). There are several common methods for encapsulating probiotics, including emulsification, extrusions, spray drying, freeze drying, and fluidized bed drying. Each approach influences the features of microencapsulated bacteria, such as their size, stability, and encapsulation efficiency (Pech‐Canul et al. 2020). Emulsification‐based encapsulation systems are appropriate ways for enhancing the water solubility and bioavailability of drugs, particularly hydrophobic compounds, and are frequently utilized in the delivery of water‐insoluble active chemicals (Boonlao et al. 2020). In unstable colloid–dispersion systems, two immiscible phases (usually water and oil) are mixed, and one liquid is dispersed in the other as small droplets, resulting in oil‐in‐water and water‐in‐oil emulsions (Ephrem et al. 2018). Emulsions systems are unstable and need to be stabilized using emulsifiers and stabilizers. Stabilizers can increase the stability of emulsions systems by increasing viscosity. Gum Arabic (GA), derived from the acacia tree, can be used as a natural emulsifier and stabilizer. Polysaccharides like GA consist of branched structures that include units of (1 → 3) and (1 → 6)‐linked β‐d‐galactopyranosyl, as well as (1 → 6)‐linked β‐d‐glucopyranosyluronic acid. GA dissolves easily in water and yields a clear solution (Ali, Ziada, and Blunden 2009). It has also been reported that gum Arabic at a concentration of 2% can lead to the stability of oil‐in‐water emulsions along with soybean protein concentrate against sodium chloride salt and high temperatures (Wang et al. 2011). Whey protein concentrate (WPC) has also been widely used in the preparation of emulsions (Setiowati, Wijaya, and Van der Meeren 2020). WPC contains up to 80% protein. This protein is also widely used in the food industry due to its emulsifying, gelling, and foaming properties (Liu et al. 2020). The current research utilized the emulsification technique, incorporating whey protein concentrate (WPC) and gum Arabic (GA) as wall materials, along with sunflower oil as the dispersed phase, to encapsulate *Lactobacillus reuteri* bacteria.

## 2 Materials and Methods

### 2.1 Material

*Lactobacillus reuteri* (IBRC‐M 10755) of human origin was provided by biological reserves center of Iran. The microcapsules were prepared in a biopolymer matrix‐based WPC and gum Arabic. Tween 80, sunflower oil, and culture mediums were of analytical grade.

### 2.2 Preparation of Microorganisms

*Lactobacillus reuteri* was cultured in MRS broth culture medium at 37°C for 48 h until 10^8^ CFU/g (half McFarland standard) were formed. Then, the biomass (bacterial cells) was separated from the culture medium by centrifugation at 5000 *g* (Hermel, Z36 HK, Germany), 25°C for 15 min, the obtained biomass was re‐suspended in 1.5 mL culture medium containing 50% glycerol and kept in −80 freezer for further analysis.

### 2.3 Microencapsulation of *Lactobacillus reuteri*

Microencapsulation was carried out using the emulsion production method with gum Arabic at concentrations of 0.5%, 1%, and 1.5%, WPC at concentrations of 1%, 2%, and 4%, and sunflower oil maintained at a constant level of 5%. Bacterial cells were inoculated in two screw‐cap jars, each of them contained 100 mL of MRS broth culture medium, and incubated at a temperature of 37°C for 48 h until reaching 10^11^ CFU/mL and then the biomass (bacterial cells) was separated from the culture medium by centrifugation at 4000 *g* (Hermel, Z36 HK, Germany), 25°C for 15 min and washed in two steps with sterile Ringer's solution (physiology serum) (Mokarram et al. 2009). One of the two groups from the previous step was diluted with 100 mL of Ringer solution to make a suspension and was considered as a control sample. The other sample was suspended in 10 mL of Ringer solution and used for encapsulation. For emulsion preparation, Tween 80 was added to sunflower oil at a ratio of 0.2 w/w and placed on a magnetic stirrer at a speed of 350 rpm for 20 min to form capsules. On the other hand, 85 mL of distilled water was poured into a screw‐cap jar and placed on a magnetic stirrer, and gum Arabic was slowly added to it. Then WPC was added to the glass and stirring was continued until the mixture was uniformed. Then 5 mL of oil containing Tween 80 (0.2% by weight) was slowly added to the mixture. The mixture was then homogenized by laboratory homogenizer (Digital Ultra Turrux T‐25; IKA instruments, Germany) at a speed of 10,000 rpm for 2 min to obtain a uniform emulsion. The emulsion was then autoclaved for sterilization and after cooling, the microbial suspension was gradually added to emulsion to complete the encapsulation process of bacterial cells. The samples were stored in the fridge for additional study (Sharifi et al. 2021).

### 2.4 Survival of Encapsulated Bacterial Cell

For counting, 1 mL of encapsulated bacteria suspension was added in 9 mL of sterile Ringer solution. The released bacteria were diluted in tubes containing sterile Ringer solution. After dilution, they were cultured using the pour plate method on solid MRS medium and incubated for 48 h at 37°C ± 1°C in an anaerobic jar (Sharifi et al. 2021).

### 2.5 Encapsulation Efficiency

1 mL of microencapsulated and control sample was diluted in 9 mL of sterile ringer solution. The encapsulated samples were stirred for 10 min at room temperature to dissolve and release the bacteria. Then, serial dilutions were cultured in MRS agar and incubated at 37°C for 48 h. After a period of time (48 h), the bacteria were counted. Three replications were done for each sample (Mokarram et al. 2009).

Finally, the encapsulation efficiency was calculated with the following formula:

$$\mathrm{EY}=N/N_{0}\times 100$$

In the above formula, EY is encapsulation efficiency, *N* is the population of living bacteria after encapsulation, and *N*_0_ is the cell population before encapsulation (Raddatz et al. 2020).

### 2.6 Heat Resistance of Encapsulated Bacterial Cells

In order to measure the heat resistance of bacteria in free and capsulated state, 1 mL of capsulated and control samples were diluted in 9 mL of sterile Ringer solution and kept at temperatures of 40°C, 60°C, 80°C, and 100°C for 30 min in a water bath. Then the viability of bacteria was determined by the method mentioned in paragraph 2.4 (Feng et al. 2018).

### 2.7 pH Resistance of Encapsulated Bacterial Cells

The pH of Ringer's solution was adjusted to 2, 4.5, and 6.5 using hydrochloric acid. 1 mL of the suspension of the capsulated and control samples were diluted in 9 mL of Ringer solution (adjusted pH) and incubated at 37°C for 3 h, then the survival of the bacteria was determined by the method mentioned in paragraph 2.4 (Silva et al. 2018).

### 2.8 Resistance of Capsulated Bacterial Cells to Bile Salts

Ringer solution containing 1% bile salt was prepared. 1 mL of the encapsulated and control samples were diluted in 9 mL of Ringer solution containing bile salt and placed in a 37°C incubator for 4 h, and then the survival of the bacteria was determined by the method mentioned in paragraph 2.4 (Silva et al. 2018).

### 2.9 Optimal Sample Selection

After conducting the above tests, it is important to highlight that the sample with the highest survival rate has been chosen for further analysis.

#### 2.9.1 Viability of Bacteria During Storage

The control and encapsulated suspension samples were stored in closed jars without controlling the presence of oxygen and humidity for 90 days at a temperature of 7°C (to simulate real conditions of cold storage). The viability of bacteria was checked on Days 0, 30, 60 and 90 through the method explained in paragraph 2.4 (Silva et al. 2018).

#### 2.9.2 Survival Rate of Encapsulated Bacteria in Simulated Conditions of Gastrointestinal

To create the simulated stomach media, sterile distilled water was used to dissolve 9 g/L of sodium chloride and 3 g/L of pepsin. The pH was then adjusted to 1.8 by adding hydrochloric acid. To create the intestinal condition, the following ingredients were dissolved in sterile distilled water: 9 g/L of sodium chloride, 10 g/L of pancreatin, 10 g/L of bovine pancreatic trypsin, and 3 g/L of bile salt. The pH of the solution was then adjusted to 5.6 using NaOH. To check the survival in digestive system conditions, 1 mL of encapsulated microbial suspension was added to 9 mL of simulated stomach media and incubated at 37°C for 60 min. Then the pH of the environment was neutralized by sodium hydroxide, and 9% of the simulated intestinal environment was added to the falcon tube and incubated for 60 min at a temperature of 37°C, and then the viability of bacteria was examined according to the method explained in paragraph 2.4, at 0, 30, 60, 90, and 120 min. This process was repeated during the storage period (3 months), with 30‐day intervals (Kazemi et al. 2022).

#### 2.9.3 Morphological Characteristics of Microcapsules

Electron microscope (Stereoscan 360; Leica, Cambridge, UK) with a voltage of 20 kW and magnification of 1000× was used to determine the morphology of encapsulated bacteria (Kazemi et al. 2022).

#### 2.9.4 Particle Size and Surface Charge (Zeta Potential) of Emulsion Droplets

Particle size distribution and average particle diameter size were measured using (DLS, Dynamic Light Diffraction) (OMEC Instrument Co. Jinding Harbor Avenue, Zhuhai, Guangdong, China) (Kazemi et al. 2022).

The zeta potential of emulsion was measured using a zeta sizer (Cordouan, VASCO 3, France) (Kazemi et al. 2022).

#### 2.9.5 Thermal Properties of Microparticles

In order to determine the thermal properties of the samples, Mettler Toledo (DSC, Differential Scanning Calorimeter) (Mettler Toledo, Schwerzenbach, Switzerland) was used. 10 mg of freeze‐dried emulsion transferred into an aluminum pan and sealed with an aluminum cover using a tablet press machine and then the temperature was raised from −50°C to 100°C at a speed of 10°C per minute under the presence of nitrogen (Kazemi et al. 2022).

#### 2.9.6 Interactions of Emulsion Components (FTIR)

In order to identify functional groups and determine the type of bonds established between emulsion components, infrared spectroscopy was used. Twenty mg of freeze‐dried emulsion was used for testing. All experiments were performed by Thermo Nicolet AVATAR 370 FTIR spectrophotometer, in the wavelength range of 350–400 cm^−1^ (Ali, Ziada, and Blunden 2009; Krithika and Preetha 2019).

### 2.10 Statistical Analysis

Statistical analysis of the samples was conducted using SPSS software (Version 26.0, IBM SPSS Inc., USA). The ANOVA test was applied to the samples and the results were presented as means ± standard deviation. Significant differences were analyzed at a *p*‐level of 0.05 using Duncan's test.

## 3 Results and Discussion

### 3.1 Encapsulation Efficiency

**FIGURE 1:**
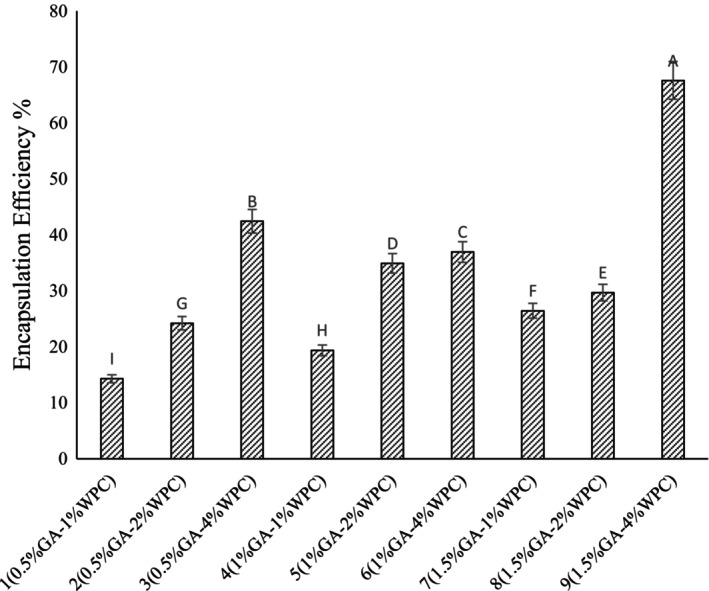
Encapsulation efficiency of *Lactobacillus reuteri* in different emulsions fabricated by WPC (1%, 2% and 4% w/v) and gum Arabic (0.5%, 1% and 1.5% w/v).

Figure 1 shows the encapsulation efficiency of *Lactobacillus reuteri* in different emulsion formulations fabricated by GA and WPC. The highest encapsulation efficiency was obtained at the highest concentration of wall materials (1.5% GA and 4% WPC) and it was reached to a maximum value of 67.57%. The encapsulation efficiency increased with increasing WPC content at the constant ratios of GA. The ability of GA‐WPC stabilized emulsions to preserve *Lactobacillus reuteri* can be attributed to the effective adsorption of two biopolymers at the oil–water interface, which effectively protected *Lactobacillus reuteri* against harsh environmental conditions (Hou et al. 2015). In the research of Raddatz et al. a high encapsulation efficiency (ranged from 91.24% to 90.59%) was observed when PRB (1% pectin + 10% rice bran) and PIN (1% pectin + 10% inulin) was used as wall material (Raddatz et al. 2020).

### 3.2 Temperature Resistance

**FIGURE 2:**
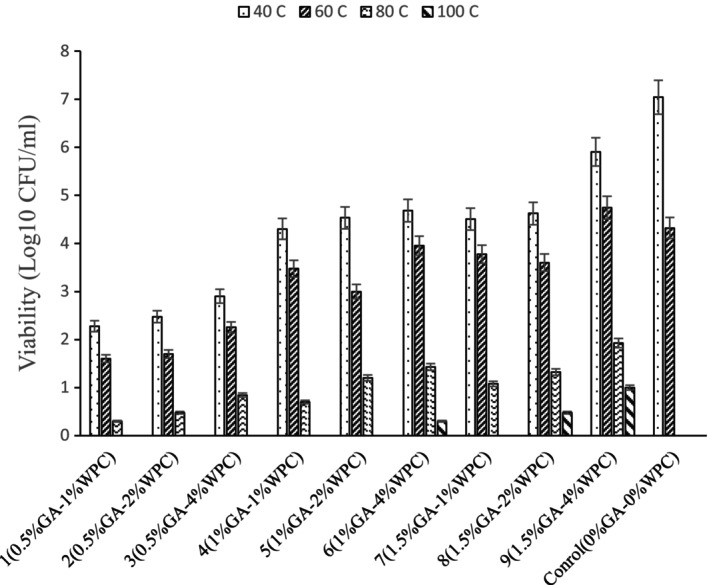
Viability of *Lactobacillus reuteri* in emulsions fabricated by WPC and GA as stabilizer at different temperature values (40°C, 60°C, 80°C, and 100°C).

The survival rates of *Lactobacillus reuteri* significantly decreased with increasing temperature in different emulsion systems (Figure 2). The viability of *Lactobacillus reuteri* in emulsion fabricated by 4% WPC and 1.5% GA was 5.90, 4.74, 1.92, and 1 Log CFU/mL at 40°C, 60°C, 80°C, and 100°C, respectively. However, the nonencapsulated cells and samples encapsulated in emulsions with WPC (< 4% w/v) and GA (< 1% w/v) lost their viability when the temperature raised to 80°C and 100°C. Emulsion systems loss their integrity and become unstable during heating. In this study, emulsions fabricated by smaller ratios of WPC and GA as wall materials showed less resistant against elevated temperatures due to weaker interfacial layer and subsequently the released bacterial cell into the medium were more susceptible to detrimental effects of heating (Kazemi et al. 2022), but emulsions at higher ratios of wall materials provide a stronger interfacial layer around oil droplets which exerted a considerable resistance against flocculation and coalescence and alleviated the heat shock to the encapsulated cells and protected them during heating. The survivability of probiotic cells against pasteurization was significantly enhanced at higher particle concentration in the high internal phase emulsion developed by Su et al. (2021) using β‐Lactoglobulin‐propylene glycol alginate. This was attributed to the higher resistance against flocculation and coalescence, resulted in a higher survival rate than the minimum effective concentration (Su et al. 2021).

### 3.3 pH Tolerance

**FIGURE 3:**
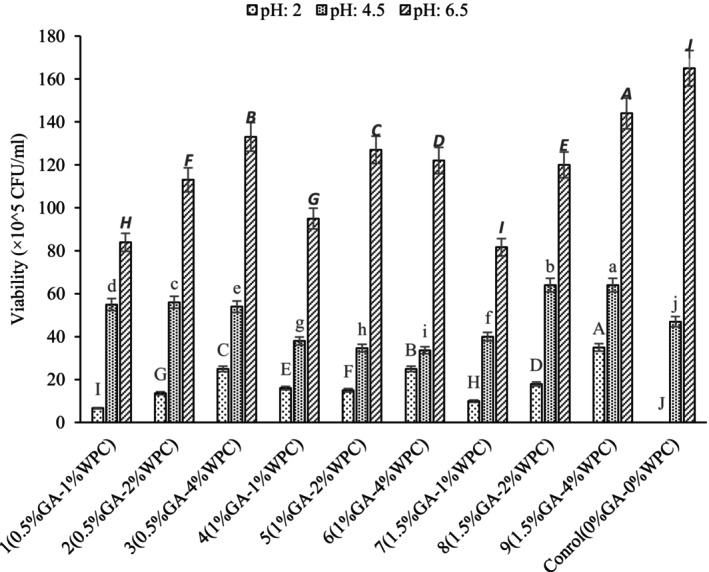
Viability of *Lactobacillus reuteri* in emulsions fabricated by WPC and GA as stabilizer at different pH values (2, 4.5, and 6.5).

The viable cell counts of *Lactobacillus reuteri* were measured after being exposed to various pH conditions (2.0, 4.5, and 6.5) for both free and encapsulated cells using emulsions fabricated with WPC and GA (Figure 3). The highest viable cell counts were observed at pH levels of 6.5, 4.5, and 2.0, respectively. No viable cells could be seen in nonencapsulated cells at pH 2.0. However, non‐encapsulated cell exhibited the highest viability at pH 6.5. It can be stated that viability of *Lactobacillus reuteri* was significantly improved after encapsulation in the emulsion at harsh pH condition (pH 4.5 and 2.0) and the viability of *Lactobacillus reuteri* was lower during storage at pH 2.0 than pH 6.5. The highest viable cell count for the encapsulated cells was 144 × 10^5^ CFU/mL in emulsion containing 4% WPC and 1.5% GA. The higher viability of *Lactobacillus reuteri* can be attributed to the emulsion structure. The thicker interfacial layer of emulsion fabricated by 4% WPC and 1.5% GA could potentially provide a barrier against droplet flocculation and coalescence which in turn provide a good protection against harsh environmental condition and increases the viability of encapsulated cells (Zhang, Lin, and Zhong 2015). Silva et al. (2018) developed two lipid microparticles to encapsulate probiotics at low pH levels. Their research showed a remarkable survival rate of over 90%, a significant improvement compared to just 55% for free probiotics. They also demonstrated the effectiveness of their encapsulation methods in protecting probiotics at pH 4.5 and 7, making them suitable for use in low pH food products.

### 3.4 Salt Tolerance

The recommended minimum amount of viable probiotic cells in food to provide health benefits to the individual is within the range of 10^6^–10^7^ colony‐forming units CFU/mL or g of food. (Feng et al. 2018).

**FIGURE 4:**
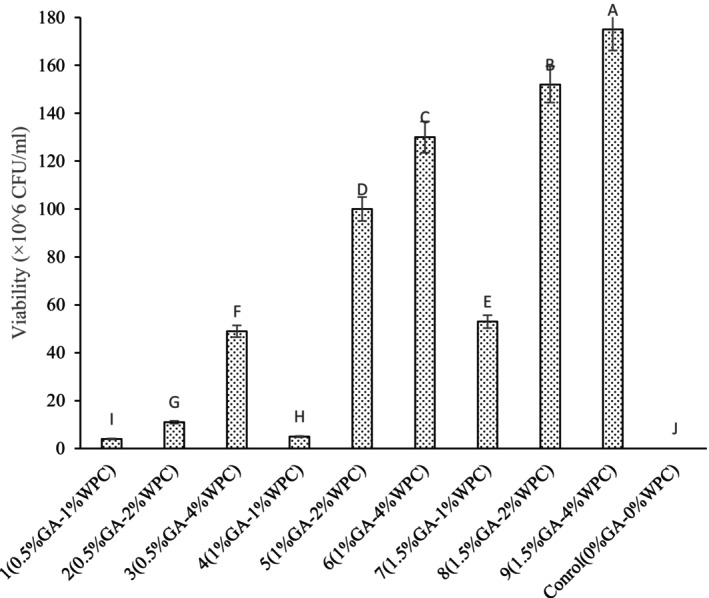
Viability of free and encapsulated *Lactobacillus reuteri* cells in different emulsion formulations after exposure to salt treatment.

As shown in Figure 4, the cell viability of *Lactobacillus reuteri* at the presence of salt increased as the ratio of WPC increased at constant ratios of GA and it was reached to its maximum value in the emulsions containing the highest concentration of wall materials. The thick layers of biopolymers in emulsion droplets can reduce the inhibitory effect of salt molecules on survival of *Lactobacillus reuteri* during storage. Similarly, Madureira et al. (2011) reported that the presence of a structured matrix, such as whey cheese, appeared to favorably influence the survival of probiotic strains during digestion (Madureira et al. 2011). Oberoi et al. (2021) demonstrated that microencapsulated *L. rhamnosus* in alginate + xanthan gum microcapsules exhibited high survivability in acidic and bile environments, maintaining good cell numbers and probiotic activity, whereas free cells decreased vitality dramatically. They also found that microencapsulated cells outlived free cells when exposed to bile, supporting the efficacy of alginate and xanthan gum as protective materials (Oberoi et al. 2021).

### 3.5 Cell Viability During Storage

**FIGURE 5:**
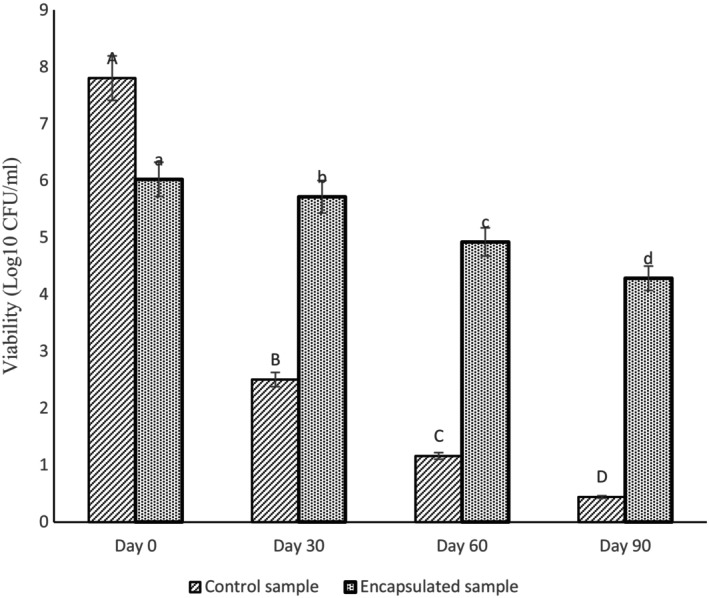
Viability of *Lactobacillus reuteri* microencapsulated with emulsion containing gum Arabic and whey protein during storage at different times.

The viability of microcapsulated *Lactobacillus reuteri* cells was evaluated at 30 days intervals during storage in the refrigerator (4°C) for 3 months. The degree of inactivation of microencapsulated bacterial cells in the optimized emulsion is presented in Figure 5. The bacterial population in the control sample decreased significantly during storage in the refrigerator. However, the microencapsulation of *Lactobacillus reuteri* cells in the dispersed phase of the GA‐WPC stabilized emulsion increased the protection of the cells, resulting in effective survival of *Lactobacillus reuteri* cells (about 6 log CFU/mL). It can be stated that the probiotic bacteria were successfully encapsulated inside the emulsion and higher survival stability was achieved during storage. Similarly, Frakolaki et al. (2021), showed that microcapsulating using a novel double emulsion effectively increased the viability of probiotics by more than ten logarithmic cycles during storage at 4°C for 4 weeks.

Iqbal et al. (2022) investigated the mono and double layered encapsulation on the stability of *Bifidobacterium bifidum* in set‐type yogurt. Double layered coating containing sodium alginate, chitosan, and whey protein concentrate achieved over 90% encapsulation efficiency and sustained probiotic viability above 10^6^ CFU/mL for 28 days compared with free cells. The findings also indicated that double layered with a polysaccharide‐protein matrix is excellent at maintaining probiotic stability in yogurt (Iqbal et al. 2022).

The survival of *Enterococcus faceium* probiotic bacteria in whey protein stabilized emulsion was monitored over a 60‐day period at 10‐day intervals. Initially, the viable count of *Enterococcus faceium* was 34 × 10^7^ CFU/mL, and it remained relatively constant for the first 30 days. By the end of the 60th day, the count had increased to 73 × 10^7^ CFU/mL (Krithika and Preetha 2019).

In a study conducted by Kheirkhahan, Ahari, and Asadi (2017), on microencapsulation of *Bifidobacterium breve* with calcium alginate and resistant corn starch by emulsification method, the number of bacteria counted for the sample containing microencapsulated *Bifidobacterium breve* after 30 days of storage at 4°C reached 8.44 ± 0.12 CFU/mL, while for the chocolate sample containing *Bifidobacterium breve* without microencapsulation, it reached 5.43 ± 0.07 CFU/mL after 30 days of storage at 4°C (Kheirkhahan, Ahari, and Asadi 2017).

### 3.6 Viability of Probiotics in the Gastrointestinal Conditions

**FIGURE 6:**
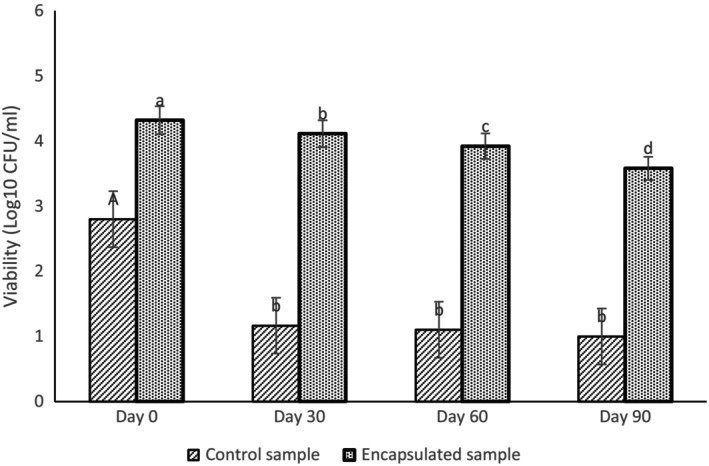
Investigating the viability of probiotics in simulated conditions of the digestive system during storage at different times.

Encapsulated bacterial cells showed higher survival in simulated conditions of stomach and intestine (Figure 6). After 90 days of storage, the number of live bacteria in the primary cells decreased from 4.32 to 3.58 Log CFU/mL, indicating a reduction of 0.74 Log CFU/mL or 82.88% of the initial bacterial population. However, for the control sample the content of primary live bacteria decreased from 2.8 to 1 Log CFU/mL after 90 days of storage. The number of living nonencapsulated bacteria in simulated stomach conditions after 30 days of storage decreased from 2.8 to 1.1 Log CFU/mL and remained constant during 60 and 90 days of storage. Krasaekoopt and Watcharapoka (2014), also showed that the microencapsulation of *Lactobacillus acidophilus* and *Lactobacillus casei* in galacto‐oligosaccharides or inulin increased their resistance to low pH and the bile salts, which is consistent with the findings of the present study (Krasaekoopt and Watcharapoka 2014). Raddatz et al. used emulsification method for microencapsulation of *Lactobacillus acidophilus* using different wall material including PEC (1% pectin), PHM (1% pectin + 10% resistant starch), PIN (1% pectin + 10% inulin), and PRB (1% pectin + 10% rice bran). They stated that all treatments provided greater protection against microorganisms after exposure to simulated gastrointestinal conditions compared with noncapsulated microorganisms, with uncoated microorganisms showing a 3.30 log reduction. But for the microorganisms covered by PEC, PHM, PIN, and PRB, a decrease of 0.11, 0.9, 1.63, and 2.37 log was observed, respectively (Raddatz et al. 2020). Rodríguez‐Huezo et al. (2014), microencapsulated *Lactobacillus plantarum* by emulsion method. Their studies showed that the acidic conditions of the stomach reduced the noncapsulated microorganisms, but the encapsulated samples showed high resistance to the acidic conditions of the stomach.

### 3.7 Morphological Characteristics of Microcapsules

**FIGURE 7:**
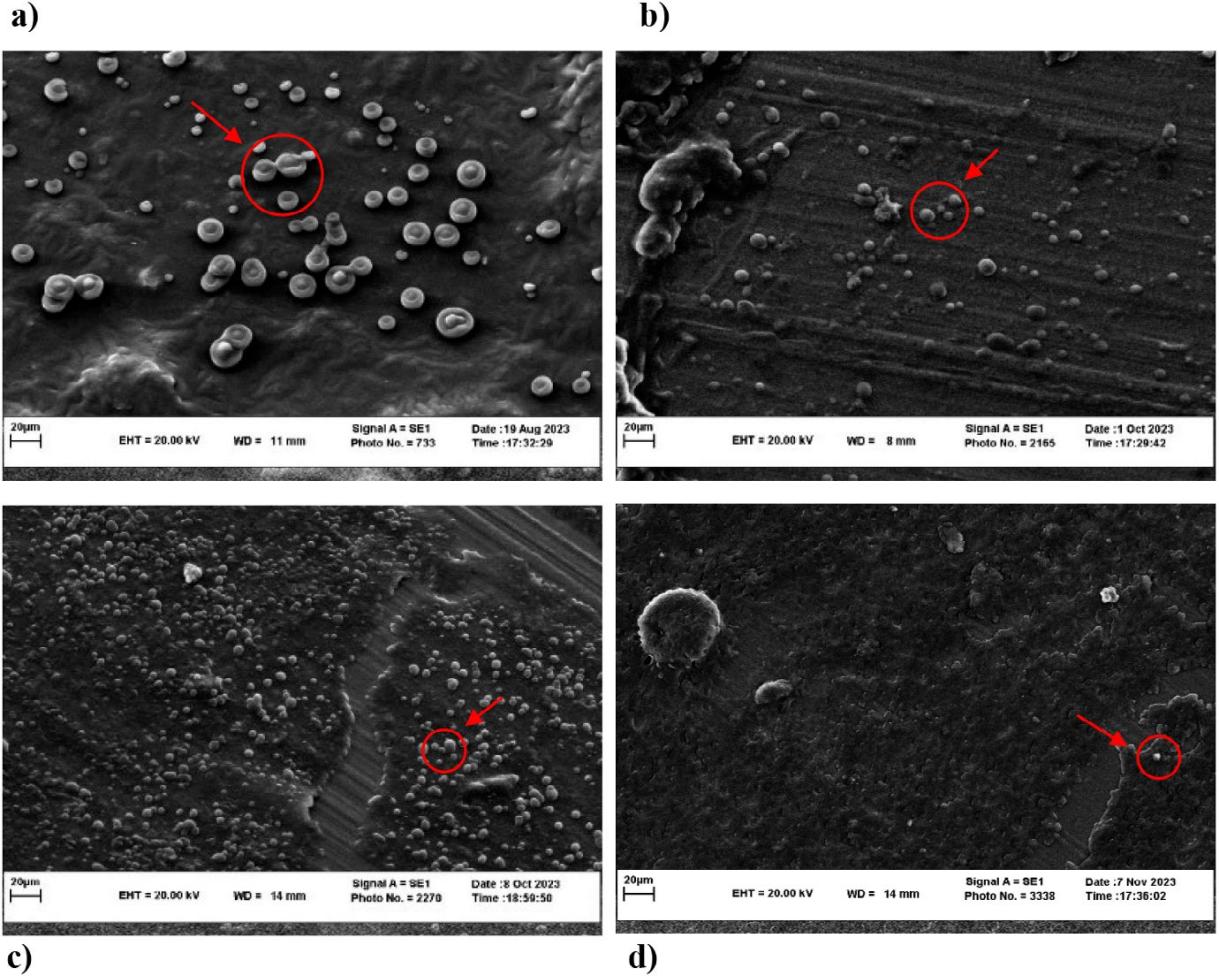
Scanning electron microscope images (magnification 1000 ×) of microencapsulated *Lactobacillus reuteri* cells in emulsion stabilized with gum Arabic and whey protein as wall materials at different storage times. The first day of storage (a), the second day of storage (b), the third day of storage (c), and the fourth day of storage (d).

Scanning electron microscope was used to investigate the changes in the morphology of emulsions containing *Lactobacillus reuteri* at different storage times. The microcapsules were spherical in shape, without any cracks or pores on the particle surfaces (Figure 7). It can be stated that the microbial cells remained inside the fresh emulsion droplets. But during storage for 30, 60, and 90 days, the emulsions become unstable and microbial cells were visible in the continuous phase of the emulsion, gradually. This instability can be attributed to factors such as phase separation and the flocculation of emulsion droplets, which led to the migration of microbial cells into the continuous phase (Zhang, Lin, and Zhong 2015).

### 3.8 Particle Size and Surface Charge (Zeta Potential) of Emulsion Droplets

Many properties of emulsions depend on particle size and particle size distribution in the emulsion. Emulsion stability and other quality indicators are therefore dependent on particle size in emulsions. The effect of storage time on particle size and surface charge (zeta potential) of optimized emulsion is shown in Table 1. During time, the droplet size of the emulsion increased in which the particle size of incubated emulsions for 90 days of storage, increased by more than two times. These results indicate the flocculation of emulsion droplets during storage. This phenomenon can be affected by the presence of non‐adsorbing polysaccharides in the emulsion, which can affect the stability of the emulsion through the flocculation of droplets. As reported by Taghrir (2020), the incubation time did not significantly affect the particle size of the emulsion when *Lactobacilli gasseri* and *Lactobacillus rhamnosus* were encapsulated in water in oil in water emulsions.

**TABLE 1:** Particle diameter and zeta potential of optimal emulsion at different storage times.

Sample	D4, 3 (μm)	Zeta potential (mV)
Day 1	2.2 ± 0.031^a^	−19.63 ± 1.23^b^
Day 30	2.6 ± 0.018^b^	−20.90 ± 4.12^a^
Day 60	3.3 ± 0.044^c^	−17.62 ± 1.88^d^
Day 90	4.6 ± 0.011^d^	−16.76 ± 1.05^c^

*Note:* Values with different superscript letters within a column are significantly different (*p* < 0.05).

Zeta potential indicates the stability of an emulsion over time. Coalescence and flocculation cause the instability of the emulsion system. These two processes change the zeta potential of the emulsion by screening the surface charge of the droplets. In this study, the incubation significantly reduced the absolute zeta potential of the optimized emulsion, and the incubated samples for 90 days showed the lowest absolute zeta potential. The decrease in surface charge indicates the weakening of the electrostatic repulsion between the emulsion droplets over time, and the flocculation of the droplets (Hou et al. 2015).

The microencapsulation of probiotics using WPC, GA, inulin, and coconut oil by nanoemulsions method was studied by (Krithika and Preetha 2019). The initial droplet size was < 150 nm, which increased to 300 and 500 nm after 30 and 60 days of storage, respectively.

### 3.9 Thermal Properties of Microcapsules (DSC)

In the DSC profiles of the samples, two transition temperatures were observed. The presence of an endothermic peak around 200°C in all samples suggests denaturation and moisture loss, as reported by (Hou et al. 2015). The melting point of gum Arabic is 85°C, while that of whey protein is 76°C (as shown in Table 2). Additionally, the second transition temperature of approximately 300°C observed in gum Arabic suggests polymerization and thermal decomposition of the gum. Whey protein exhibits two distinct transition temperatures: approximately 76°C and 295°C. The first transition temperature corresponds to the amorphous phase, which is associated with the evaporation of residual moisture within the overlapping protein structure. The second transition, occurring around 300°C, signifies the melting of the crystalline portion. In the emulsion samples, the phase change temperature was observed to be higher than 200°C, indicating an enhancement in the thermal resistance of capsules. This improvement can be attributed to the energy changes resulting from electrostatic interactions between the two wall materials—gum Arabic and whey protein. Notably, microcapsules exhibited a broader endothermic peak compared to gum Arabic and whey protein powders, as reported by (Hou et al. 2015).

**TABLE 2:** Thermal properties of samples during storage.

Arabic gum	Whey protein	Day 1	Day 30	Day 60	Day 90
Δ*H*1 = 48 Δ*H*2 = 52	Δ*H*1 = 35 Δ*H*2 = 32	Δ*H*1 = 55 Δ*H*2 = 20	Δ*H*1 = 57 Δ*H*2 = 23	Δ*H*1 = 32 Δ*H*2 = 21	Δ*H*1 = 33 Δ*H*2 = 22
*T*_s_ = 67 *T*_m_ = 85	*T*_s_ = 64 *T*_m_ = 76	*T*_s_ = 203 *T*_m_ = 226	*T*_s_ = 211 *T*_m_ = 227	*T*_s_ = 211 *T*_m_ = 226	*T*_s_ = 35 *T*_m_ = 74
*T*_s_ = 270 *T*_m_ = 298	*T*_s_ = 281 *T*_m_ = 295	*T*_s_ = 261 *T*_m_ = 283	*T*_s_ = 324 *T*_m_ = 346	*T*_s_ = 265 *T*_m_ = 284	*T*_s_ = 212 *T*_m_ = 228

*Note:* Δ*H* is enthalpy, *T*_s_ and *T*_m_ represent melting and degradation temperature, respectively.

A significant decrease was observed for initial phase change temperature in the sample stored for 90 days. This decline suggests phase separation and overall instability of the emulsion during the storage period.

### 3.10 FTIR Spectroscopy

**FIGURE 8:**
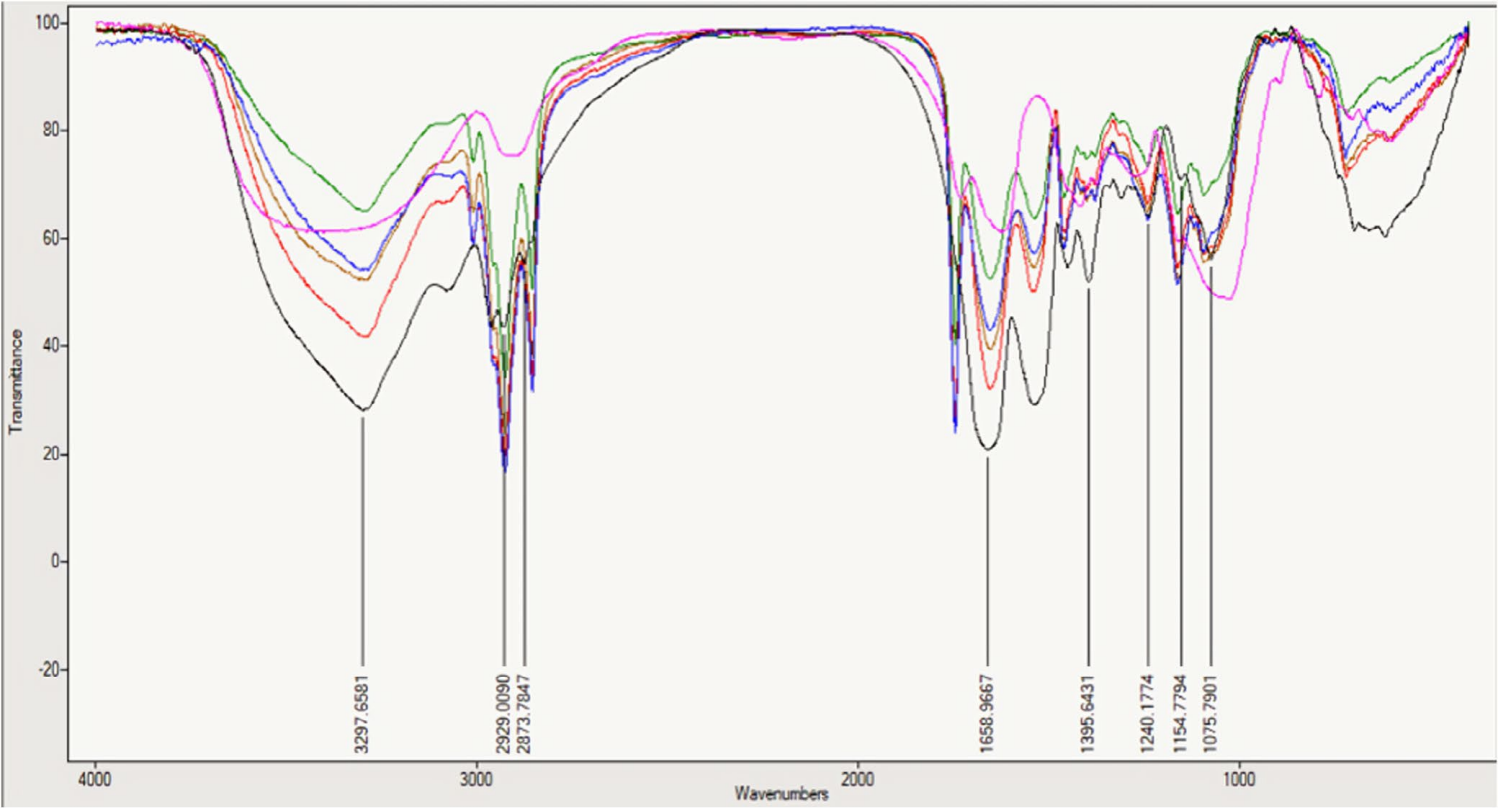
FTIR spectra of raw materials (gum Arabic and whey protein) microcapsules during different storage times.

Figure 8 displays the FTIR spectra of the raw materials (gum Arabic and whey protein concentrate) as well as the microcapsules at various storage times. In all samples, the stretching vibrations of OH groups were observed as a broad absorption spectrum at 3381 cm^−1^. Additionally, the microcapsulated samples exhibited strong absorption bands at 2854 and 2925 cm^−1^, corresponding to the symmetric and asymmetric stretching vibrations of CH_₂_ groups. In the case of microcapsules, specific regions corresponding to amide I (C=O stretching), amide II (N–H bending), and amide III (C–N stretching and N–H deformation) exhibited peaks at wave numbers 1655, 1537, and 1240 cm^−1^, respectively, within the whey protein structure (as reported by Bhagwat et al. 2020). Additionally, the observed peak at wave number 1745 cm^−1^ is attributed to the −C=O bond in the ester structure. In the nanocapsules, a prominent band at 1544 cm^−1^ (associated with amide II) was observed. This band corresponds to the –C–N– stretching vibration and the angle stretching involving N–H in the structure of –C=ONH– (also referred to as acylamino‐II) (Hou et al. 2015). Furthermore, a novel absorption peak at 1746 cm^−1^ emerged in the WPC/GA nanocapsules, confirming the electrostatic interaction between the amine groups (−NH₃^+^) of WPC and the carboxyl groups (–COO^−^) of GA. The FTIR spectrum showcases gum Arabic samples (pink), whey protein (black), Day 0 (brown), Day 30 (green), Day 60 (red), and Day 90 (blue) samples.

## 4 Conclusion

The research findings demonstrated that the stability and quality of emulsion droplets are influenced by their size and surface charge (zeta potential). Over time, the droplet size increased, indicating flocculation during storage, potentially influenced by nonadsorbing polysaccharides. Emulsion stability can be affected by phenomena such as flocculation and droplet aggregation, leading to changes in zeta potential and weakening of electrostatic repulsion between droplets. The highest encapsulation efficiency was achieved with 1.5% gum Arabic and 4% whey protein concentrate, likely due to effective adsorption of the two polymers at the oil–water interface. Emulsions containing these concentrations increased the survival of encapsulated *Lactobacillus reuteri*, even in harsh conditions like the presence of salt, low pH, and high temperature, indicating a protective effect of the emulsion structure. Electron microscope images revealed instability of the emulsions after 30, 60, and 90 days of storage, but microbial cells remained visible in the continuous phase, possibly influenced by factors such as phase separation and accumulation of emulsion droplets over time. Storage of optimized emulsions for 3 months at 4°C showed increased survival of *Lactobacillus reuteri* cells. During the 90‐day storage period, there was a significant decrease in the survival of microencapsulated bacteria in emulsions under gastrointestinal conditions. The presence of amide groups in the protein structure was confirmed by FTIR spectroscopy, which also indicated electrostatic interactions between gum Arabic and whey protein, supporting the formation of microcapsules. The findings of this study demonstrated that the encapsulation of *Lactobacillus reuteri* bacteria using the emulsification method with gum Arabic and whey proteins effectively preserves the probiotic bacteria in the food substrate during storage. Therefore, they are ideal for creating frozen probiotic products like ice cream, cultured products such as yogurt and cheese, as well as acidophilus milks and other fermented dairy drinks.

## Author Contributions

**Forough Teymoori:** conceptualization (equal), formal analysis (equal), methodology (equal), software (equal), writing – original draft (equal). **Sahar Roshanak:** investigation (equal), visualization (equal), writing – review and editing (equal). **Shadi Bolourian:** funding acquisition (equal), writing – review and editing (equal). **Rassoul Mozafarpour:** formal analysis (equal), investigation (equal), methodology (equal), writing – review and editing (equal). **Fakhri Shahidi:** funding acquisition (equal), supervision (equal), writing – original draft (equal), writing – review and editing (equal).

## Ethics Statement

The authors have nothing to report.

## Conflicts of Interest

The authors declare no conflicts of interest.

## Data Availability

All data generated or analyzed during this study are included in this published article.
